# Early Hypothalamic Aromatase Loss Is Associated with Neuronal Insulin-Resistance Signaling During Progression of Experimental Alzheimer’s Disease

**DOI:** 10.3390/biology15211970

**Published:** 2026-11-02

**Authors:** Yujiao Lu, Sagar Vyavahare, Mayuri Gulhane, Xinxin Dong, Abbas Jarrahi, Harminder D Singh, Molly Braun, Darrell W. Brann, Krishnan M. Dhandapani

**Affiliations:** Department of Neurosurgery, Medical College of Georgia, Augusta University, Augusta, GA 30912, USA; svyavahare@augusta.edu (S.V.); mgulhane@augusta.edu (M.G.); xidong@augusta.edu (X.D.); ajarrahi@augusta.edu (A.J.); hsingh2@augusta.edu (H.D.S.); mobraun@augusta.edu (M.B.); dbrann@augusta.edu (D.W.B.)

**Keywords:** Alzheimer’s disease, dementia, brain aromatase, estrogen, insulin resistance

## Abstract

Alzheimer’s disease (AD) is increasingly recognized as a neurodegenerative disorder characterized by central insulin resistance and cerebral hypometabolism. The steroid hormone, 17β-estradiol (E2), interacts closely with insulin signaling pathways and is locally synthesized in the brain by the enzyme aromatase. However, the spatial and temporal dynamics of brain aromatase during AD progression and its relationship to central insulin resistance remain poorly understood. Using the 5xFAD mouse model, we examined whole-brain, regional, and neuron/astrocyte-specific alterations in aromatase expression along disease progression. Whole-brain aromatase expression remained unchanged at 2 months of age but progressively declined at 5 and 9 months, inversely correlating with amyloid-β plaque burden. Regional analysis of the cortex, hippocampal CA1, and hypothalamus revealed progressive aromatase loss in all regions; notably, the hypothalamus exhibited a significant decline at the earliest disease stage examined, preceding comparable changes in the cortex and hippocampus, while an age-related decline cannot be fully excluded, as only a single-aged wild-type (WT) control was used. Cell-level analysis revealed a progressive reduction in neuronal aromatase expression, whereas astrocytic aromatase displayed a biphasic response, increasing during early disease stages before declining at later stages. In parallel, neuronal insulin-resistance signaling increased progressively with disease advancement and was negatively correlated with neuronal aromatase expression across all regions examined (r = −0.82 to −0.87). Collectively, these findings demonstrate progressive regional and neuron/astrocyte-specific alterations in brain aromatase during the progression of amyloid pathology, identifying early hypothalamic vulnerability as a feature of AD-associated altered insulin signaling.

## Simple Summary

Alzheimer’s disease (AD) is associated with brain insulin resistance and progressive metabolic dysfunction. Although brain-derived estrogen has well-established neuroprotective properties, it remains unclear how the estrogen-synthesizing enzyme, aromatase, changes during AD progression and whether these changes are associated with central insulin resistance. Using 5xFAD mice, we found that aromatase expression progressively declined across multiple brain regions and neuronal populations as amyloid pathology progressed. Notably, the hypothalamus exhibited aromatase loss at an earlier stage than the cortex and hippocampus, identifying this metabolically important brain region as particularly vulnerable during early progression of AD. In contrast to the progressive reduction in neurons, an early surge followed by a later-stage decline in aromatase was observed in reactive astrocytes. We further observed that neuronal insulin-resistance signaling was strongly associated with neuronal aromatase loss. Together, these findings reveal distinct regional and neuron/astrocyte-specific changes in brain aromatase during AD progression and highlight early hypothalamic dysfunction as a potential feature of AD-associated metabolic impairment.

## 1. Introduction

Alzheimer’s disease (AD) is the most common cause of dementia and is characterized by extracellular amyloid-β (Aβ) plaques, intracellular neurofibrillary tangles, synaptic dysfunction, and progressive cognitive decline [1,2,3,4]. Increasing evidence suggests that metabolic dysfunction is an early feature of AD and may contribute to disease progression [5,6,7]. Notably, cerebral hypometabolism can be detected years before the onset of clinical symptoms, indicating impaired brain metabolism is not simply a downstream consequence of established neuropathology [8,9].

Brain insulin resistance is increasingly recognized as a key component of AD pathophysiology and has led to the characterization of AD as a form of “type 3 diabetes” [8,9,10]. Under physiological conditions, insulin signaling regulates glucose transport and utilization, mitochondrial function, and synaptic plasticity through activation of the insulin receptor (IR), insulin receptor substrate (IRS), and downstream PI3K/AKT and MAPK pathways [11,12]. Disruption of these signaling pathways impairs neuronal metabolism and is associated with Aβ accumulation, tau hyperphosphorylation, oxidative stress, and neuroinflammation, all of which contribute to neurodegenerative processes [13,14,15].

17β-estradiol (E2) is a neuroactive steroid with well-established neuroprotective properties in both experimental and clinical studies of neurological disease, including AD [16,17,18,19]. Emerging evidence indicates substantial crosstalk between estrogen and insulin signaling pathways. E2 enhances insulin sensitivity through modulation of insulin receptor signaling, mitochondrial bioenergetics, and glucose metabolism [20,21], whereas estrogen deficiency is associated with impaired insulin responsiveness and reduced cerebral glucose utilization [8,22]. These findings suggest that alterations in brain estrogen signaling may contribute to metabolic dysfunction during AD progression.

Although E2 is traditionally considered a peripheral hormone produced primarily by the ovaries, it is also synthesized locally within the brain by aromatase. Aromatase is expressed in the brains of both males and females and, under physiological conditions, is predominantly localized to neurons throughout multiple regions, including the cortex, hippocampus, hypothalamus, and amygdala [23,24,25]. Locally synthesized estrogen functions as an important neuromodulator that regulates synaptic plasticity, cognitive processes, and cerebral metabolism [26,27,28,29]. Following brain injury, however, aromatase is robustly induced in reactive astrocytes, where it contributes to neuroprotective and reparative responses [30,31,32]. Clinical and experimental studies further suggest that reduced brain aromatase expression may be associated with neuronal vulnerability and AD-related pathology [22,23,33].

Despite growing evidence supporting neuroprotective roles for brain-derived estrogen, the spatial and temporal dynamics of aromatase expression during AD progression remain poorly defined. In particular, it is unclear whether region-specific and neuron-specific alterations in aromatase are associated with the emergence of neuronal insulin resistance. Therefore, the present study used the 5xFAD mouse model to characterize whole-brain, regional, and neuron/astrocyte-specific changes in aromatase expression throughout progression of amyloid pathology and to examine their relationship with neuronal insulin-resistance signaling.

## 2. Materials and Methods

### 2.1. Experimental Animals

Male C57BL/6J and 5xFAD transgenic mice were used in this study. 5xFAD mice were purchased from Jackson Laboratory, and genotypes were confirmed by genotyping protocols (034840-JAX). The primer sequences used for genotyping were: mutant reverse, CGG GCC TCT TCG CTA TTA C; wild-type reverse, TAT ACA ACC TTG GGG GAT GG; and common, ACC CCC ATG TCA GAG TTC CT. Wild-type (WT) controls were C57BL/6J mice assessed at 3 months of age, purchased from Jackson Laboratory (JAX:000664). Animals were housed under controlled conditions (22–24 °C, 50–60% humidity) on a 12 h light:12 h dark cycle with ad libitum access to food and water. Mice were randomized into four experimental groups: wild-type (WT) controls (3 months of age) and 5xFAD mice at 2, 5, and 9 months of age (*n* = 5). Sample size was determined a priori based on the primary outcome measure [34]. Pre-specified exclusion criteria included behavioral abnormalities, body weight loss of >15%, or severe ulcerative dermatitis; however, no animals met these criteria during the course of the study, and therefore none were excluded. All mice were maintained in the same environment to minimize potential confounders. Investigators performing the experimental procedures were aware of group allocation; however, investigators performing outcome assessments and data analyses were blinded to group allocation until completion of the analyses. All animal procedures were performed in accordance with the National Institutes of Health Guide for the Care and Use of Laboratory Animals and were approved by the Augusta University Institutional Animal Care and Use Committee (IACUC).

### 2.2. Preparation of Brain Sections and Histological Analysis

Brain tissue collection and processing were performed as previously described [34]. Briefly, mice were deeply anesthetized and transcardially perfused with ice-cold 0.9% saline followed by 4% paraformaldehyde (PFA). Brains were removed, post-fixed in 4% PFA at 4 °C for 48 h, and cryoprotected in 30% sucrose until tissues sank completely. Samples were embedded in OCT compound and sectioned coronally at 25 μm thickness using a Thermo Scientific MICROM HM 550 cryostat (Thermo Fisher Scientific, Kalamazoo, MI, USA). Regions of interest (cortex, hippocampus, and hypothalamus) were selected using consistent anatomical landmarks based on a standard mouse brain atlas. For hypothalamic assessments, images were specifically acquired within the ventromedial hypothalamic nucleus (VMH, −1.70 mm to −1.90 mm caudal to Bregma), given its established role in neuroendocrine and metabolic regulation.

The following primary antibodies were used for immunofluorescent staining: anti-aromatase (Invitrogen, Carlsbad, CA, USA, Cat# PA1-16532, 1:200), anti-β-amyloid (BioLegend, San Diego, CA, USA, Cat# 835104, 1:200), anti-NeuN (Proteintech, Rosemont, IL, USA, 66836-1-Ig, 1:500), anti-GFAP (Invitrogen, Cat# PA5-18598, 1:800), anti-phospho-IRS1 (p-IRS1^S616^) (Abcam, Waltham, MA, USA, Cat# AB4776, 1:200), and anti-IRS1 (Invitrogen, Cat# 700662, 1:300). All these antibodies have been validated by previous studies [35,36,37,38,39,40].

Fluorescent images were acquired using a Leica STELLARIS confocal microscope (Leica Microsystems, Boston, MA, USA) with either a 10× dry objective or 40× oil immersion objective. All images were collected using identical acquisition settings within each experiment and exported at a resolution of 1024 × 1024 pixels. Image analysis was performed using LAS X software (Leica Microsystems, Boston, MA, USA) and ImageJ (Fiji 2.16.0, NIH, Bethesda, MD, USA).

For quantitative analysis, integrated density (IntDen) values were measured and expressed as arbitrary units (A.U.). Fluorescence intensity measurements were normalized to cell-free background fluorescence to permit comparisons among experimental groups. For β-amyloid staining, total plaque area was quantified using ImageJ. For p-IRS1 (S616)/IRS1 normalization, IRS1 intensities in each group were first converted to a fold change relative to their WT controls. Subsequently, intensities of p-IRS1 (S616) were normalized to their corresponding fold change parameter as the normalized p-IRS1 (S616) values. All analyses were performed using unmodified images. To prevent progressive neuronal loss in 5xFAD mice from confounding our cell-specific expression analyses, quantification was performed on a per-cell basis rather than by total field area. Within each region of interest, 5 morphologically intact NeuN-positive (or GFAP-positive) cells were randomly selected per section. Fluorescence intensities for aromatase and p-IRS^S616^ were measured specifically within the boundaries of these selected cells. The values of these 5 cells were averaged to generate a single section value, and 4–5 anatomically matched sections were averaged to generate the final biological replicate value per animal. Representative images are shown in the figures.

### 2.3. Correlation Analysis

Pearson correlation analysis of individual animals was performed to assess the relationship between amyloid-β plaque burden and aromatase expression, as well as between neuronal aromatase expression and neuronal insulin-resistance signaling. Prior to analysis, assumptions of linearity and normality were evaluated using scatterplot inspection and Shapiro–Wilk testing. Correlation analyses were conducted using GraphPad Prism 11, and statistical significance was defined as *p* < 0.05.

### 2.4. Statistical Analysis

Data are presented as mean ± SEM. For quantitative analysis of immunostaining, values of multiple randomly selected sections were averaged per animal prior to statistical testing. Statistical analyses were performed using GraphPad Prism 11 (GraphPad Software, San Diego, CA, USA). Differences among multiple groups were assessed using one-way analysis of variance (ANOVA) followed by Tukey’s multiple-comparisons test when appropriate. For regional comparisons of brain aromatase, neuronal aromatase, and neuronal p-IRS1 changes in 5xFAD mice, the intensities were normalized to the mean values of their corresponding brain regions of WT mice and expressed as percentages of WT controls (WT = 100%). The effects of brain region (cortex, hippocampus, and hypothalamus), age (2, 5, and 9 months), and their interaction were evaluated using a linear mixed-effects model, with brain region, age, and their interaction as fixed effects and individual mouse as a random effect to account for repeated measurements across brain regions. Post hoc comparisons among brain regions within each age group were performed with adjustment for multiple comparisons. Statistical significance was defined as *p* < 0.05. Statistical values, including F-values, Degrees of Freedom, and *p*-values were summarized in Table S1. To account for the inflation of Type I error resulting from running multiple distinct ANOVA families, a global False Discovery Rate (FDR) correction was applied across all primary ANOVA *p*-values using the Benjamini–Hochberg procedure. For ANOVA models that remained statistically significant after FDR adjustment, Tukey’s honestly significant difference (HSD) post hoc test was performed to evaluate pairwise differences.

## 3. Results

### 3.1. Aromatase Is Progressively Reduced in 5xFAD Brains and Negatively Associated with Amyloid-β Pathology

**Figure 1:**
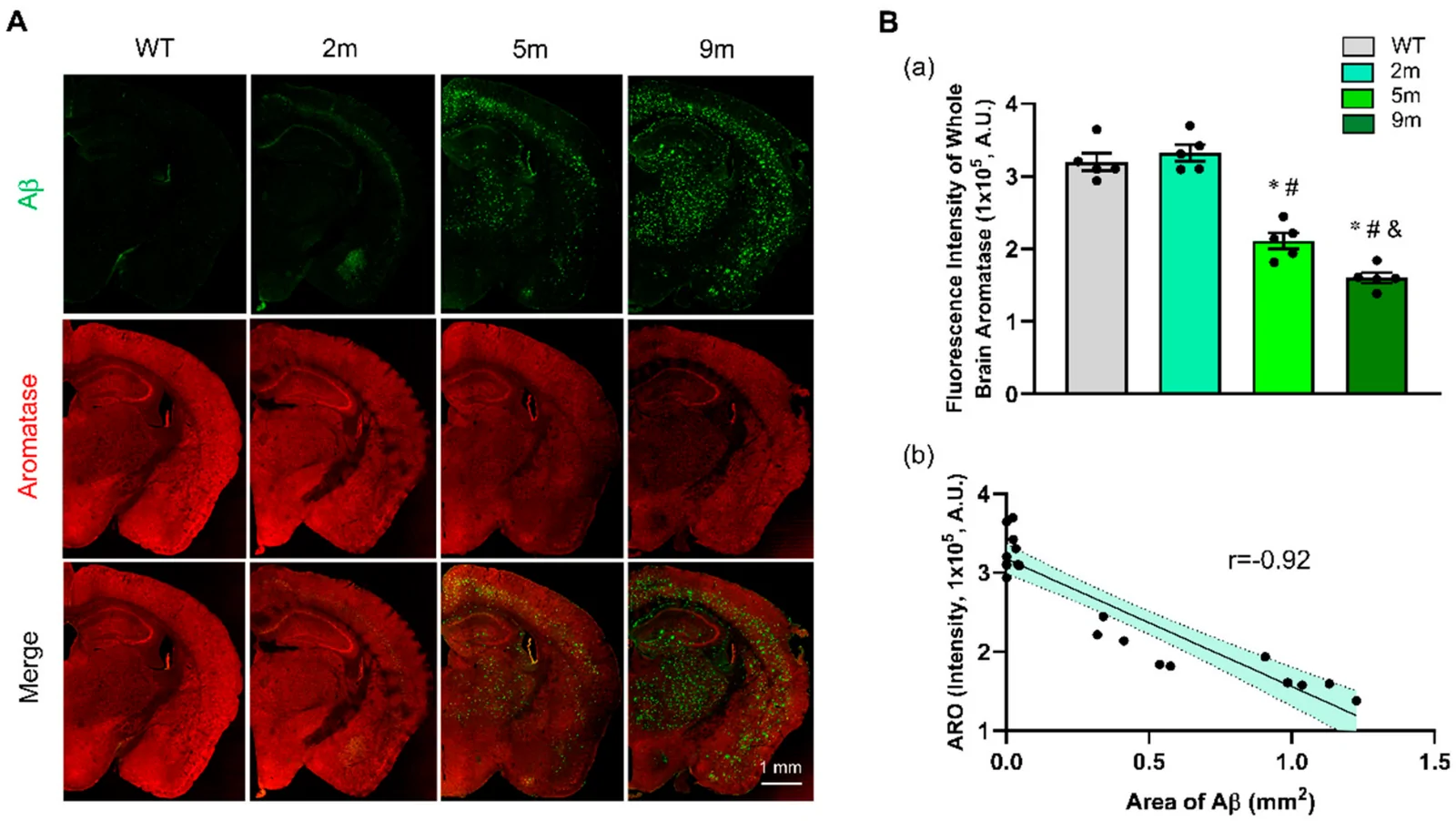
Progressive alterations of whole-brain aromatase and its correlation with beta-amyloid pathology. (**A**) Double immuno-staining of amyloid-β and aromatase in the brains of wild-type (WT, 3-month), 2-month, 5-month, and 9-month-old 5xFAD mice. (**B**) Quantitative analysis of the aromatase fluorescence intensity in the whole brain (**a**). Correlation analysis was performed to examine the linear relationship between aromatase expression and total amyloid-β area in the brains, with r value shown and the 95% confidence interval indicated in the shaded area (**b**). Values are means ± SEM from each group. *N* = 5. * *p* < 0.05 vs. WT, ^#^ *p* < 0.05 vs. 2-month, ^&^ *p* < 0.05 vs. 5-month. ARO, aromatase. A.U., arbitrary unit.

Reduced aromatase expression has been reported in brain regions affected by AD and has been associated with decreased local estrogen production and cognitive impairment [33]. To determine how aromatase expression changes during pathology progression, aromatase and Aβ double immunostaining was performed in brains from WT, 2-month, 5-month, and 9-month-old 5xFAD mice (Figure 1). As expected, Aβ immunoreactivity was absent in WT mice and progressively increased with age in 5xFAD mice (Figure 1A). Quantification of whole-brain aromatase expression revealed no significant changes at 2 months compared to controls but decreased at 5 and 9 months (Figure 1B). Correlation analysis demonstrated a strong negative relationship between whole-brain aromatase expression and Aβ plaque burden (r = −0.92; Figure 1B). Collectively, these findings indicate that whole-brain aromatase expression progressively declines during AD progression and is inversely associated with increasing amyloid pathology.

### 3.2. Region-Specific Alterations in Brain Aromatase Reveal Early Hypothalamic Vulnerability

**Figure 2:**
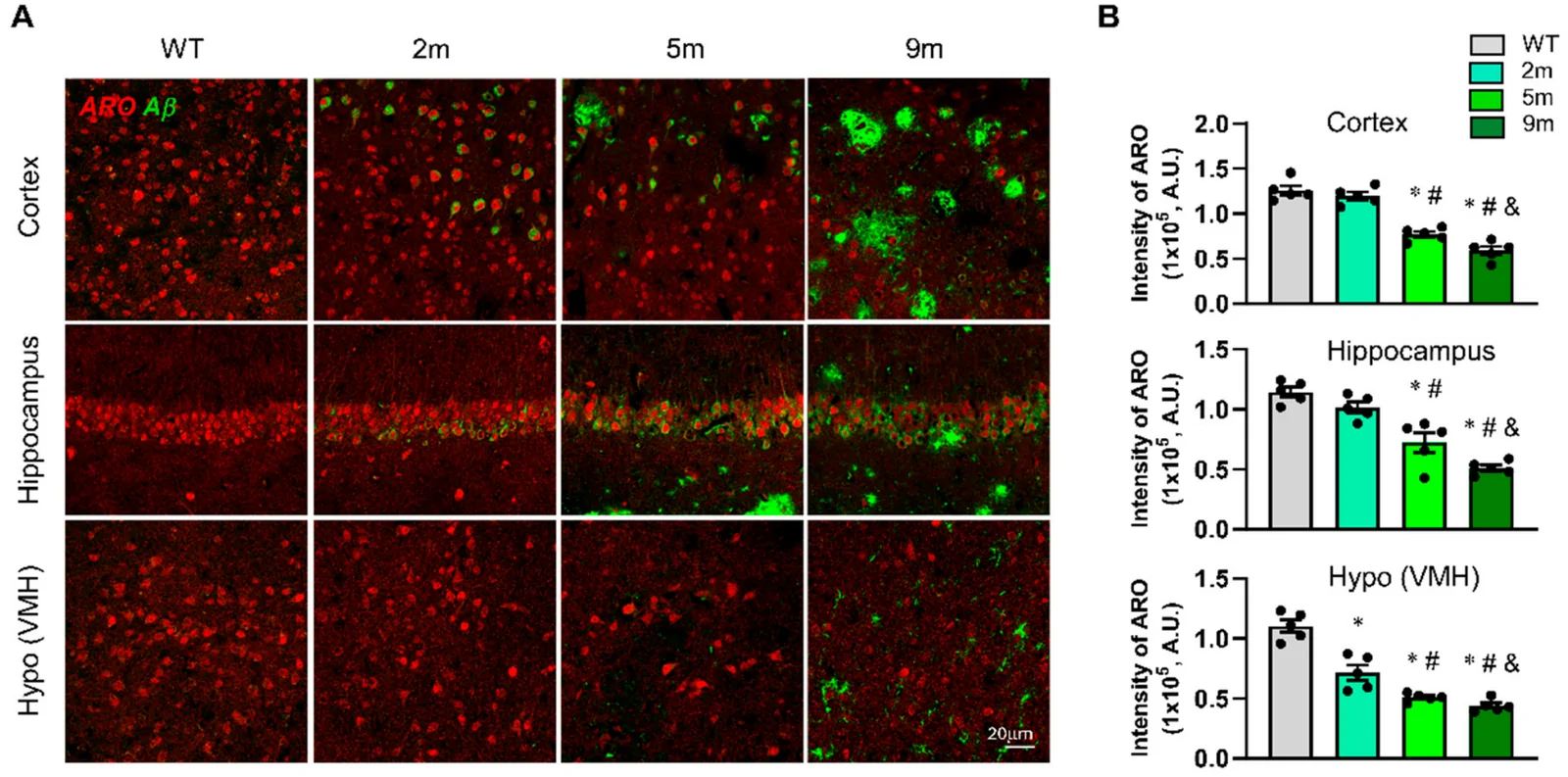
Regional analysis of brain aromatase in 5xFAD mice. (**A**) IHC double staining of aromatase and amyloid-β in the selected key brain regions, including cortex, hippocampal CA1, and hypothalamus (VMH). (**B**) Analysis and comparison of aromatase fluorescence intensities in these brain regions. Values are means ± SEM from each group. *N* = 5. * *p* < 0.05 vs. WT (3-month), ^#^ *p* < 0.05 vs. 2-month, ^&^ *p* < 0.05 vs. 5-month. ARO, aromatase. Hypo, hypothalamus. Aβ, amyloid-beta. A.U., arbitrary unit. WT, wild-type.

To determine whether aromatase expression exhibits regional differences during AD progression, aromatase immunoreactivity was evaluated in the cortex, hippocampal CA1 region, and hypothalamus (Figure 2A). Quantitative analysis revealed no significant changes in aromatase expression within the cortex or hippocampal CA1 region at 2 months of age, compared with controls (Figure 2B). In contrast, aromatase expression was significantly reduced in the hypothalamus at this early time point (Figure 2B). At 5 and 9 months, aromatase expression progressively declined across all three regions (Figure 2B). Notably, the hypothalamus was the only region exhibiting significant aromatase loss at the earliest disease stage examined, whereas cortical and hippocampal aromatase expression remained preserved. These findings identify a clear regional difference in the temporal pattern of aromatase loss and suggest that the hypothalamus is particularly affected during early AD progression.

### 3.3. Progressive Neuronal Aromatase Loss Occurs Earlier in the Hypothalamus

**Figure 3:**
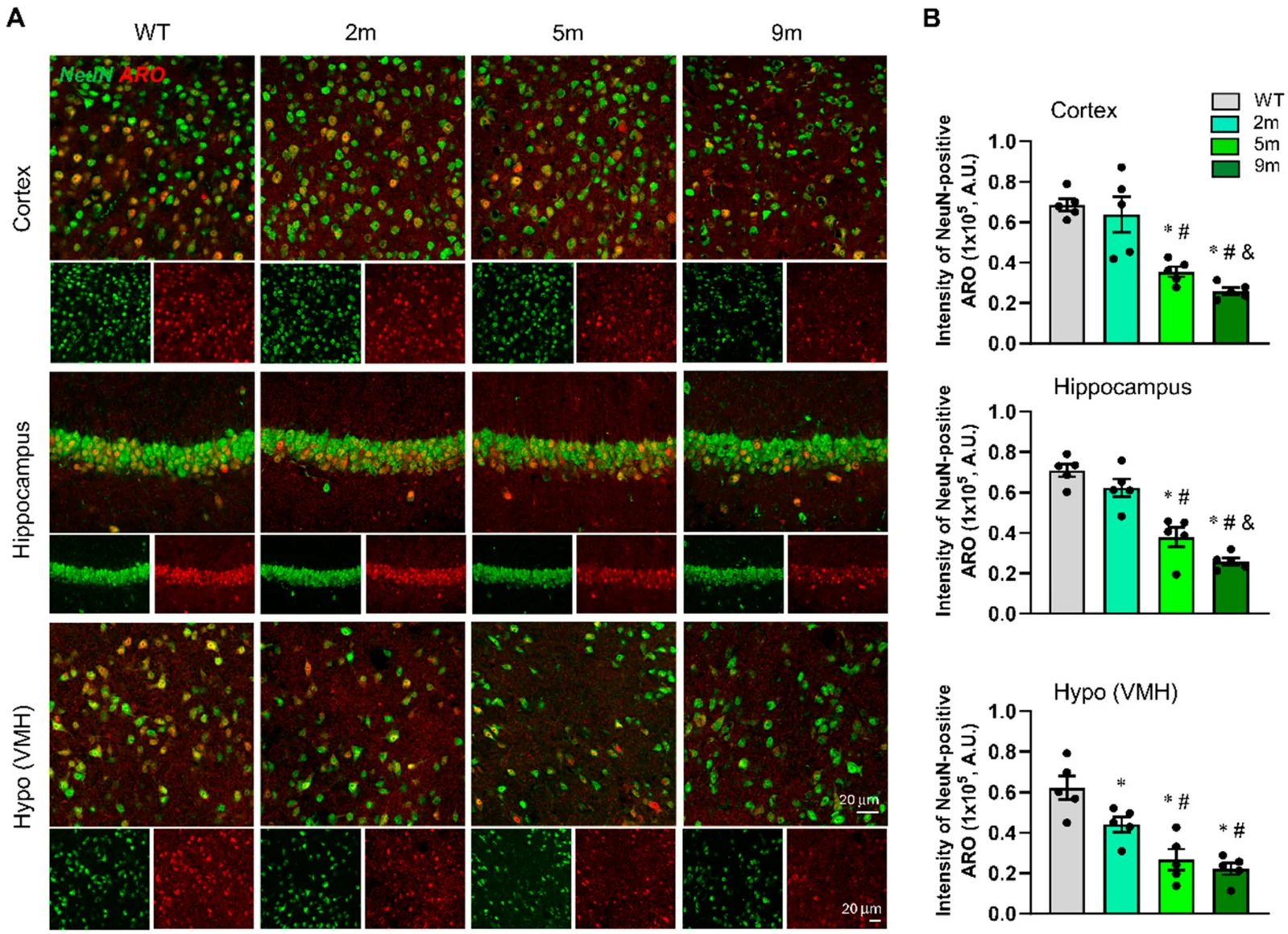
Spatial and temporal changes in neuronal aromatase in 5xFAD mice. (**A**) IHC double staining of NeuN, a neuron-specific marker, and aromatase in cortex, hippocampal CA1, and hypothalamus (VMH) of all the experimental groups. (**B**) Quantitative analysis and comparisons of aromatase intensities in NeuN-positive cells. Values are means ± SEM from each group. *N* = 5. * *p* < 0.05 vs. WT (3-month), ^#^ *p* < 0.05 vs. 2-month, ^&^ *p* < 0.05 vs. 5-month. ARO, aromatase. Hypo, hypothalamus. A.U., arbitrary unit. WT, wild-type.

Because aromatase is predominantly expressed in neurons under physiological conditions, neuronal aromatase expression was examined by NeuN/aromatase co-localization analysis (Figure 3A). Quantification revealed no significant changes in neuronal aromatase expression within the cortex or hippocampal CA1 region of 2-month-old 5xFAD mice compared with controls (Figure 3B). In contrast, hypothalamic neurons exhibited a significant reduction in aromatase expression at this early stage (Figure 3B). Neuronal aromatase expression progressively declined in the cortex and hippocampus at 5 and 9 months, with the lowest levels observed at 9 months (Figure 3B). Although hypothalamic neuronal aromatase was reduced at both 5 and 9 months, no further decrease was observed between these time points (Figure 3B). Overall, these findings demonstrate progressive neuronal aromatase loss during progression of amyloid pathology and identify the hypothalamus as the earliest region exhibiting neuronal aromatase deficiency.

### 3.4. Astrocytic Aromatase Exhibits a Biphasic Response During AD Progression

**Figure 4:**
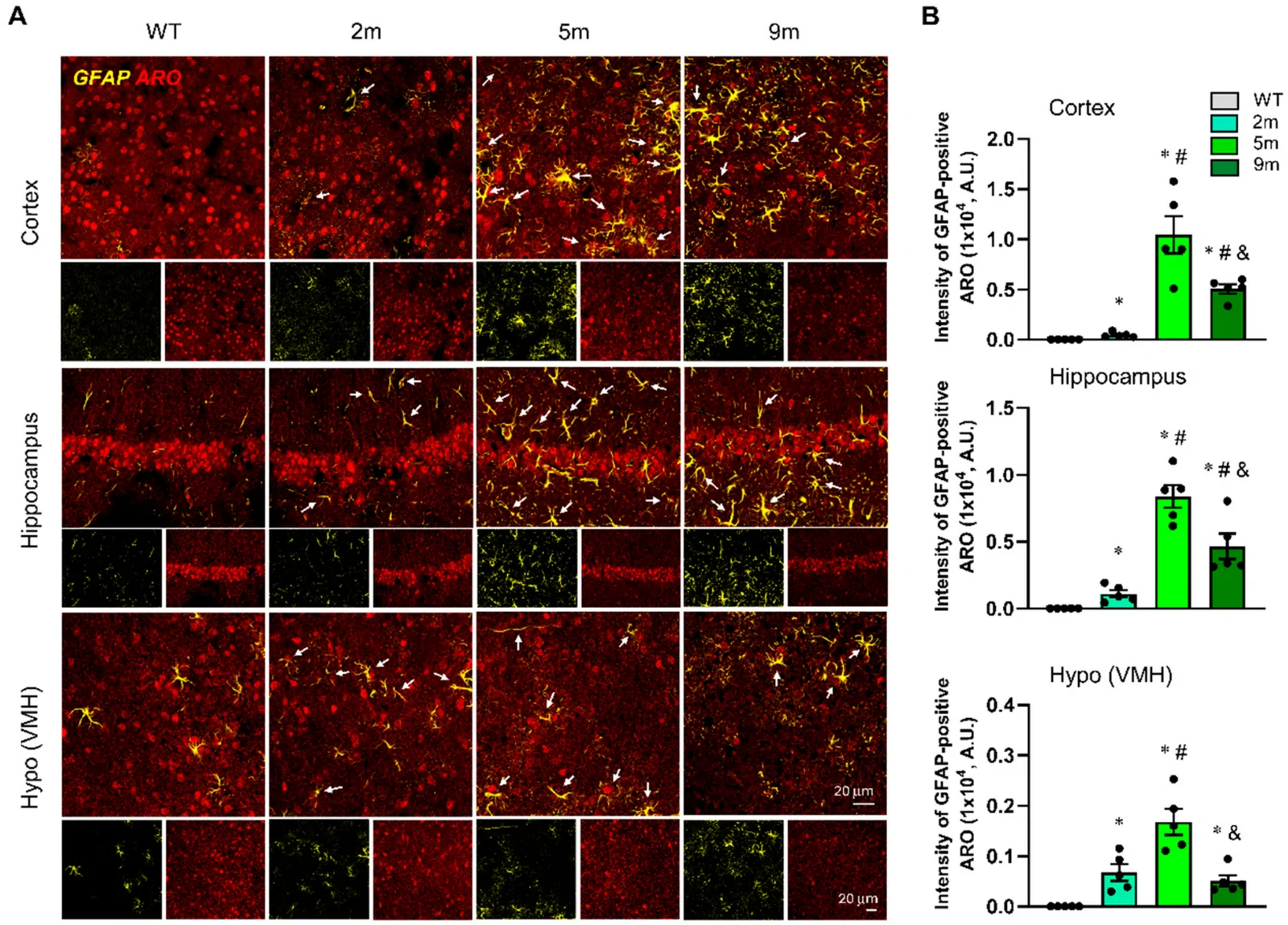
Induction and alterations of aromatase expression in astrocytes. (**A**) IHC double staining of GFAP, an astrocyte-specific marker, and aromatase in cortex, hippocampal CA1, and hypothalamus (VMH) of all the experimental groups. (**B**) Quantitative analysis and comparisons of aromatase intensities in GFAP-specific cells (aromatase-expressing astrocytes were indicated with white arrows). Values are means ± SEM from each group. *N* = 5. * *p* < 0.05 vs. WT (3-month), ^#^ *p* < 0.05 vs. 2-month, ^&^ *p* < 0.05 vs. 5-month. ARO, aromatase. Hypo, hypothalamus. A.U., arbitrary unit. WT, wild-type.

To assess the changes in astrocytic aromatase expression, GFAP/aromatase co-immunostaining was performed in the cortex, hippocampal CA1 region, and hypothalamus (Figure 4A). Consistent with previous reports, little to no aromatase expression was detected in astrocytes from control mice. In 5xFAD mice, aromatase expression increased in reactive astrocytes across all regions examined compared to controls (Figure 4A). Quantitative analysis revealed modest but significant increases in astrocytic aromatase within the cortex and hippocampus at 2 months, whereas a more robust increase was observed in the hypothalamus (Figure 4B). Astrocytic aromatase expression further increased at 5 months in all regions but, interestingly, declined significantly by 9 months (Figure 4B). Together, these findings indicate that astrocytic aromatase displays a biphasic response during AD progression, characterized by early induction followed by a decline at later disease stages.

### 3.5. Early Hypothalamic Insulin-Resistance Signaling Parallels Aromatase Loss

**Figure 5:**
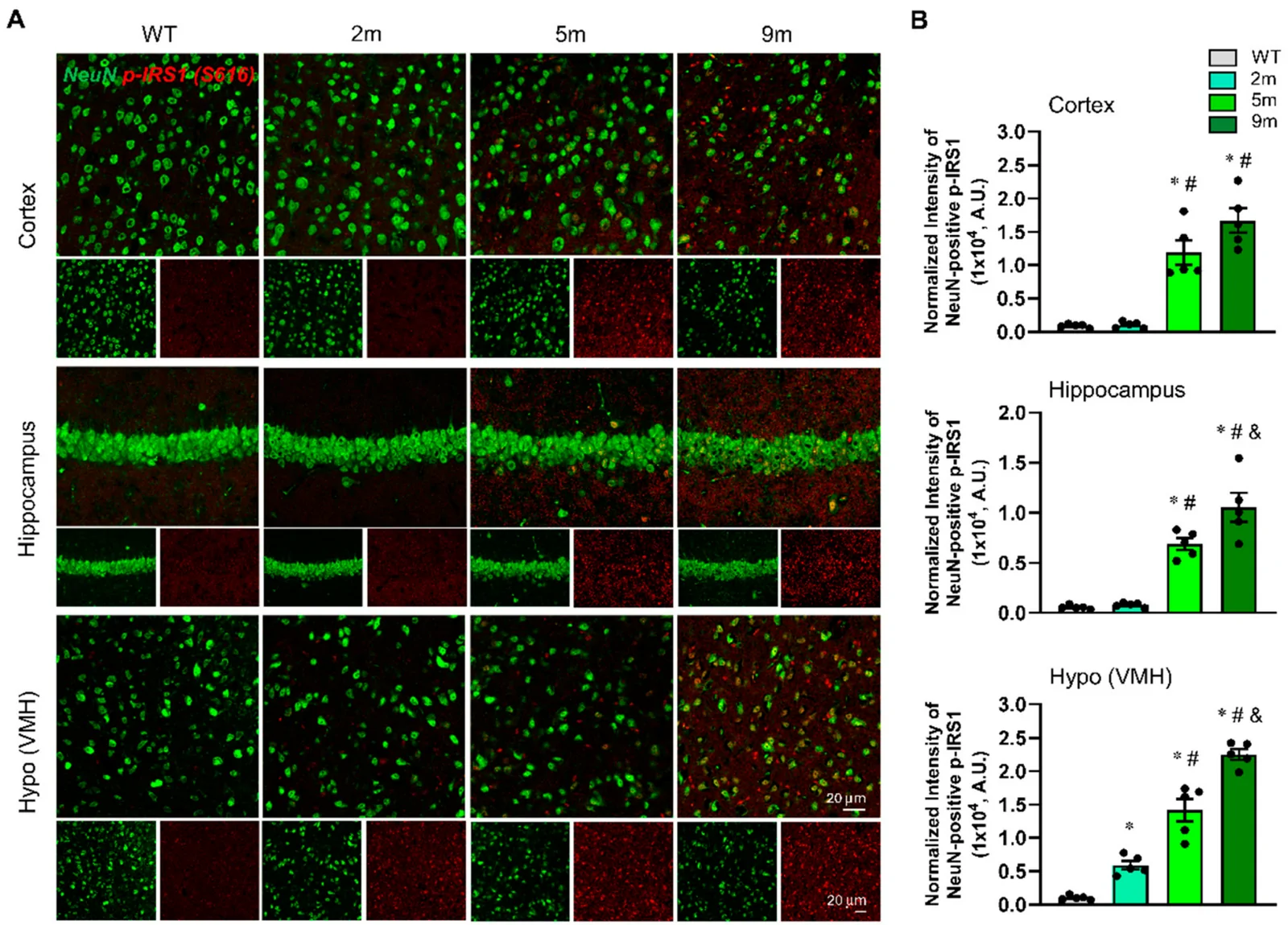
Progressive development of neuronal insulin-resistance signaling in 5xFAD mice. (**A**) IHC double staining of NeuN, a neuron-specific marker, and p-IRS1^S616^, a marker of insulin-resistance signaling, was conducted in the cortex, hippocampal CA1, and hypothalamus (VMH). (**B**) Analysis and comparison of normalized NeuN-positive p-IRS1^S616^ in these brain regions among the experimental groups. Values are means ± SEM from each group. *N* = 5. * *p* < 0.05 vs. WT (3-month), ^#^ *p* < 0.05 vs. 2-month, ^&^ *p* < 0.05 vs. 5-month. ARO, aromatase. Hypo, hypothalamus. A.U., arbitrary unit. WT, wild-type.

To determine whether neuronal insulin-resistance signaling develops during disease progression, NeuN and p-IRS1^S616^ co-immunostaining was performed (Figure 5A). Phosphorylation of IRS1 at Ser616 disrupts normal insulin signaling and is associated with AD pathophysiology; therefore, p-IRS1^S616^ serves as a marker of insulin-resistance signaling. Intensities of p-IRS1^S616^ were normalized to total IRS1, which was examined by NeuN and IRS1 double staining (Supplemental Figure S1). Quantitative analysis revealed no significant increase in neuronal p-IRS1^S616^ within the cortex or hippocampal CA1 of 2-month 5xFAD mice compared with controls (Figure 5B). In contrast, neuronal p-IRS1^S616^ expression was significantly elevated in the hypothalamus at this early time point (Figure 5B). Increased neuronal p-IRS1^S616^ immunoreactivity was subsequently observed in all examined regions at 5 and 9 months (Figure 5B). Notably, the temporal pattern of neuronal insulin-resistance signaling closely paralleled that observed for neuronal aromatase loss, with the hypothalamus exhibiting the earliest alterations. These findings demonstrate progressive development of neuronal insulin-resistance signaling during AD progression and identify the hypothalamus as an early site of altered insulin signaling.

### 3.6. Neuronal Aromatase Loss Is Strongly Associated with Insulin-Resistance Signaling

**Figure 6:**
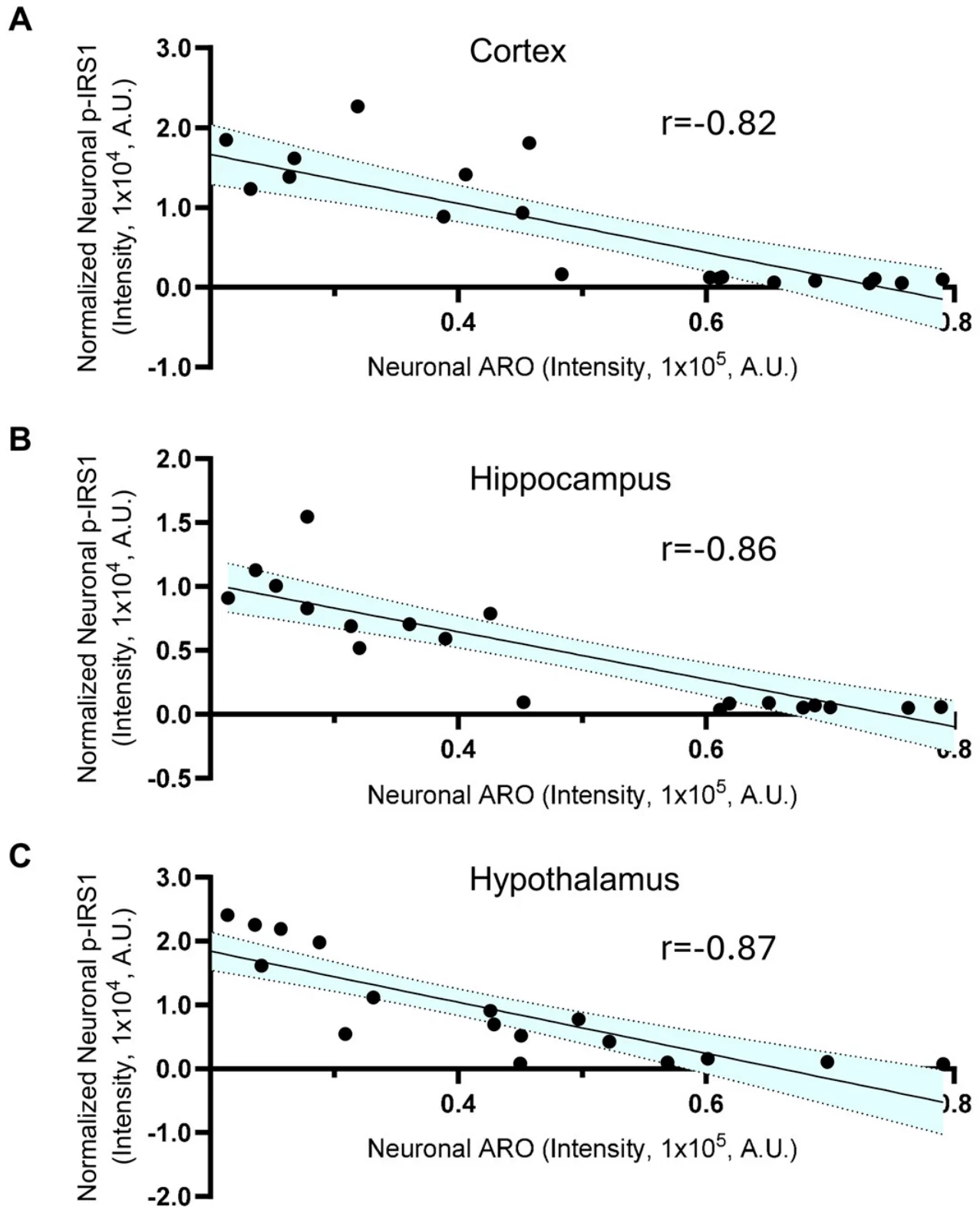
Correlation analysis of insulin-resistance signaling and aromatase alterations in neurons across both wild-type and 5xFAD cohorts. (**A**) Analysis of the linear relationship between normalized neuronal p-IRS1^S616^ signaling and neuronal aromatase intensities in the cortex of WT (3-month) and 5xFAD mice, with the correlation coefficient r value shown. (**B**) Correlation analysis of normalized neuronal p-IRS1^S616^ and aromatase intensities in hippocampal CA1, with the correlation coefficient value r shown. (**C**) Correlation analysis of normalized neuronal p-IRS1^S616^ and aromatase intensities in the hypothalamus, with the r value shown and the 95% confidence interval indicated in the shaded area. *N* = 20. ARO, aromatase. A.U., arbitrary unit.

Correlation analyses for WT and 5xFAD mice were performed to determine the relationship between neuronal aromatase expression and neuronal insulin-resistance signaling across brain regions. In the cortex, neuronal aromatase levels were strongly and negatively correlated with neuronal p-IRS1^S616^ expression (r = −0.82; Figure 6A). Similar negative correlations were observed in the hippocampus (r = −0.86; Figure 6B) and hypothalamus (r = −0.87; Figure 6C). Correlation analysis in only 5xFAD mice was also performed to examine the linear relationship under AD pathology (Supplemental Figure S2). The results suggested similar negative correlations in these brain regions (r = −0.75 for cortex, r = −0.81 for hippocampus, and r = −0.82 for hypothalamus). These findings demonstrate a robust association between reduced neuronal aromatase expression and increased neuronal insulin-resistance signaling across multiple brain regions. However, the directionality and mechanistic basis of this relationship remain to be determined.

## 4. Discussion

The present study identifies early hypothalamic aromatase loss as a previously underappreciated feature of AD pathology in 5xFAD mice. Three major findings emerged from this work. First, aromatase expression progressively declined at the whole-brain, regional, and neuronal levels as disease advanced. Second, astrocytic aromatase exhibited a biphasic response characterized by early induction followed by a decline at later stages. Third, the hypothalamus demonstrated early aromatase loss and early neuronal insulin-resistance signaling before comparable changes were detected in the cortex or hippocampus. Collectively, these observations identify region- and neuron/astrocyte-specific alterations in aromatase during AD progression and highlight the hypothalamus as a potentially early and vulnerable site of AD-associated metabolic dysfunction.

Brain insulin resistance is increasingly recognized as a central feature of AD and has contributed to the concept of AD as a form of “type 3 diabetes” [41,42,43,44]. Impaired insulin signaling disrupts cerebral glucose utilization and has been linked to amyloid accumulation, tau pathology, oxidative stress, and neuroinflammation [45,46,47,48,49]. Importantly, insulin can maintain high levels of insulin-degrading enzyme (IDE), which plays a critical role in regulating amyloid-β clearance [50]. Brain insulin resistance lowers IDE levels, reducing the degradation of amyloid-β and worsening insulin-resistance signaling [51]. Consistent with these observations, we found progressive increases in neuronal p-IRS1^S616^ immunoreactivity across all brain regions examined. Notably, hypothalamic neurons exhibited elevated p-IRS1^S616^ at the earliest disease stage analyzed, whereas similar changes were not observed in the cortex or hippocampus until later stages. These findings suggest that metabolic dysfunction may emerge earlier in the hypothalamus than in traditional cognitive regions affected by AD.

Accumulating evidence indicates that estrogen regulates insulin sensitivity, promotes glucose utilization, and protects against the development of insulin resistance [21,52,53,54]. In addition to its endocrine functions, estrogen is synthesized locally within the brain by aromatase and serves important neuromodulatory and neuroprotective roles [26,55,56,57,58,59]. Clinical and experimental studies have increasingly implicated aromatase in AD pathogenesis. Aromatase polymorphisms have been associated with increased risk of AD, and reduced aromatase expression has been reported in post-mortem AD brain tissues [60,61,62]. Furthermore, genetic ablation of aromatase in transgenic AD mice results in greater brain estrogen loss, increased Aβ deposition, and accelerated pathological progression [61]. Consistent with these reports, we observed progressive reductions in aromatase expression during AD progression. Importantly, this decline occurred earlier in the hypothalamus than in the cortex or hippocampus, identifying a distinct regional pattern of vulnerability.

The most notable finding of the present study was the identification of early hypothalamic aromatase loss during AD progression. The hypothalamus plays a central role in energy balance, glucose homeostasis, endocrine regulation, and stress responses. Although AD research has traditionally focused on the cortex and hippocampus because of their roles in learning and memory, increasing evidence suggests that hypothalamic dysfunction may also contribute to disease progression [63,64,65,66]. In the current study, both aromatase loss and neuronal insulin-resistance signaling were detected in the hypothalamus before comparable changes emerged in the cortex or hippocampus. The results were validated by both temporal changes in these brain regions and direct regional comparisons at the key pathological endpoints (Supplemental Figure S3). These observations identify a previously underappreciated regional difference in AD progression and raise the possibility that alterations in hypothalamic estrogen signaling may be associated with early metabolic abnormalities during disease development. Notably, both aromatase loss and neuronal insulin-resistance signaling emerged in the hypothalamus before detectable changes were observed in the cortex or hippocampus, suggesting that hypothalamic dysfunction may represent an early component of AD-associated metabolic disturbances.

Not all studies have reported reduced aromatase expression in AD. For example, increased aromatase immunoreactivity has been observed in the hippocampal CA4 region of AD patients [67]. The reasons underlying these apparent discrepancies remain unclear but may reflect differences in disease stage, brain regions analyzed, patient populations, or methodological approaches. Interestingly, that study also reported evidence of reduced aromatase expression in other brain regions, including the hypothalamus, which is consistent with our findings. Together, these observations suggest that aromatase alterations are region-specific and may vary across the course of disease progression.

An additional finding was the biphasic regulation of astrocytic aromatase. Consistent with previous studies of acute brain injury, we observed increased aromatase expression in reactive astrocytes during early disease stages. Interestingly, this response diminished at later stages despite marked astrocyte activation. Although the mechanisms underlying this decline remain unknown, these findings suggest that astrocytic aromatase responses may change over the course of disease progression. Future studies will be needed to determine whether this transition reflects altered astrocyte phenotypes, changes in local inflammatory signaling, or other adaptive processes.

We also observed strong negative correlations between neuronal aromatase expression and neuronal p-IRS1^S616^ levels across all brain regions examined. These findings are consistent with previous evidence demonstrating reciprocal interactions between estrogen signaling and insulin sensitivity. However, the present study was not designed to establish causality, and several interpretations remain possible. Reduced aromatase expression may contribute to impaired insulin signaling, insulin resistance may suppress aromatase expression, or both changes may arise from common downstream pathological processes. Mechanistic studies will be required to distinguish these possibilities.

Several limitations should be acknowledged. First, the findings were generated in the 5xFAD model and should be validated in additional models of AD. Second, the study is descriptive and establishes associations rather than causal relationships between aromatase loss and neuronal insulin resistance. Third, aromatase expression was assessed primarily by immunofluorescence, and future studies incorporating transcriptomic, biochemical, and functional approaches will be important for defining underlying mechanisms. Moreover, WT control mice were assessed at a single age, rather than at matched ages (2, 5, and 9 months) with 5xFAD groups. An age-related decline in aromatase cannot be fully excluded. Finally, only male mice were examined. Furthermore, because this study focused strictly on central nervous system pathology, systemic metabolic parameters and circulating hormone levels (such as testosterone) were not evaluated. Given that brain aromatase expression in males can be influenced by circulating androgens, future studies incorporating parallel assessments of systemic endocrine status will be necessary to determine the extent to which peripheral hormonal fluctuations contribute to the observed decline in local brain aromatase. A previous study in rodents suggested that males show higher baseline aromatase activity in several hypothalamic regions than females [68], whereas similar evidence in humans, especially AD brains, is still limited. However, since women bear a disproportionately higher clinical burden of AD, an interesting prediction is that female hypothalamic aromatase could be more strongly coupled to reproductive aging and estrogen status and may lose compensatory capacity during AD progression. Therefore, additional studies are needed to determine whether similar patterns occur in females given the well-established interactions between estrogen signaling and AD risk.

## 5. Conclusions

Using a 5xFAD mouse model, we identified progressive region- and neuron/astrocyte-specific alterations in brain aromatase expression during disease progression. Aromatase levels declined at the whole-brain and neuronal levels, whereas astrocytic aromatase exhibited an early upregulation followed by a later decline. Importantly, the hypothalamus demonstrated significant aromatase loss and increased neuronal insulin-resistance signaling at an earlier disease stage than the cortex or hippocampus, identifying a distinct pattern of regional vulnerability. Neuronal aromatase expression was strongly and negatively associated with neuronal p-IRS1^S616^ levels across all regions examined. Together, these findings highlight early hypothalamic dysfunction as a feature of AD progression and support future studies examining the relationship between brain-derived estrogen signaling and metabolic impairment in AD.

## Data Availability

Data are contained within the article and will be available upon request.

## Supplementary Materials

The following supporting information can be downloaded at https://www.mdpi.com/article/10.3390/biology15211970/s1. Figure S1: Progressive changes of neuronal IRS1 in 5xFAD mice; Figure S2: Correlation analysis of neuronal insulin resistance signaling and aromatase alterations in 5xFAD mice; Figure S3: Comparisons of aromatase and p-IRS1 changes among different brain regions; Table S1: Summary of statistical values.

## Abbreviations

The following abbreviations are used in this manuscript:

AD	Alzheimer’s disease
E2	17β-estradiol
CNS	Central nervous system
Aβ	Amyloid-β
IR	Insulin receptor
IRS	Insulin receptor substrate
A.U.	Arbitrary unit
WT	Wild type
PFA	Paraformaldehyde
VMH	Ventromedial hypothalamic nucleus
