# Supplementary material for: Early Hypothalamic Aromatase Loss Is Associated with Neuronal Insulin-Resistance Signaling During Progression of Experimental Alzheimer’s Disease
Source: Biology (Basel). 2026 Nov 2;15(21):1970. doi: 10.3390/biology15211970 (PMC13762160; doi:10.3390/biology15211970)
Supplement: Supplementary file 1 [file biology-15-01970-s001.zip › biology-4586461-supplementary.pdf]

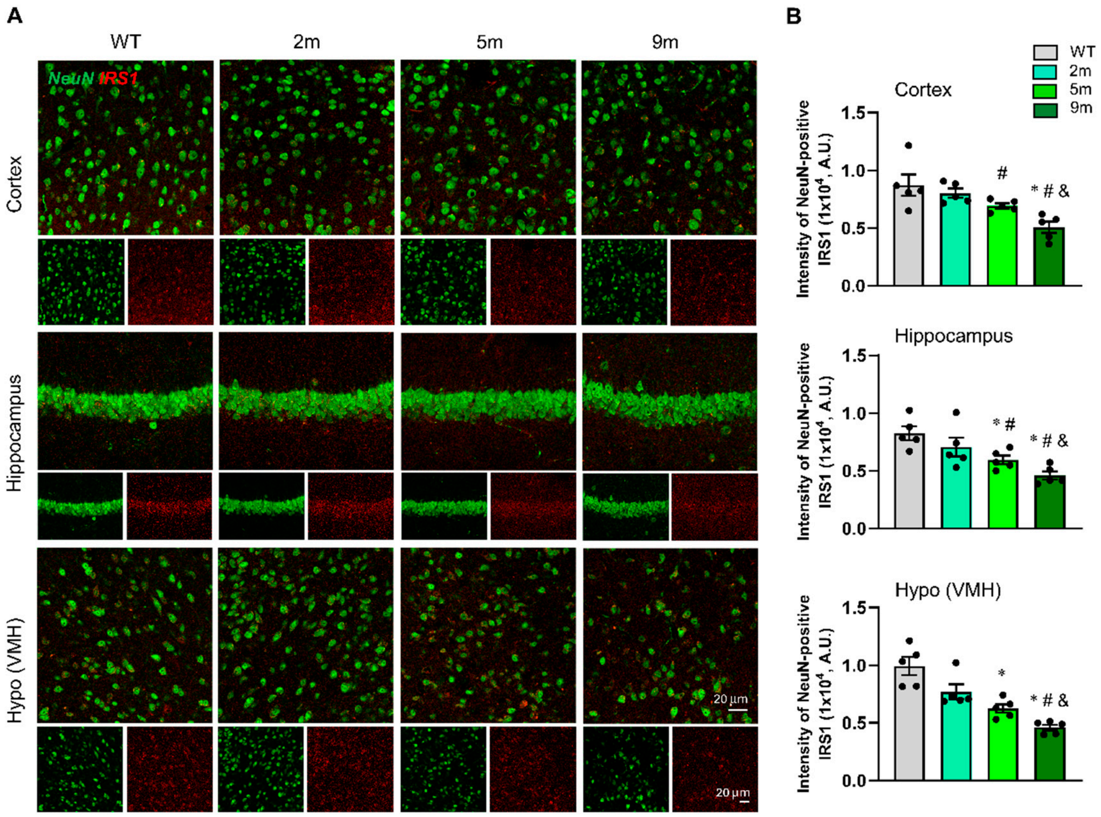

Figure S1. Progressive changes of neuronal IRS1 in 5xFAD mice. (**A**) IHC double staining of NeuN, a neuron-specific marker and IRS1, a key component of insulin signaling was conducted in cortex, hippocampal CA1 and hypothalamus (VMH). (**B**) Analysis and comparison of NeuN-positive IRS1 in these brain regions among the experimental groups. Values are means ± SEM from each group. $N = 5$. $^{*}P<0.05$ vs. WT (3-month), $^{\#}P<0.05$ vs. 2-month, $^{\&}P<0.05$ vs. 5-month. ARO, aromatase. Hypo, Hypothalamus. A.U., arbitrary unit. WT, wild-type.

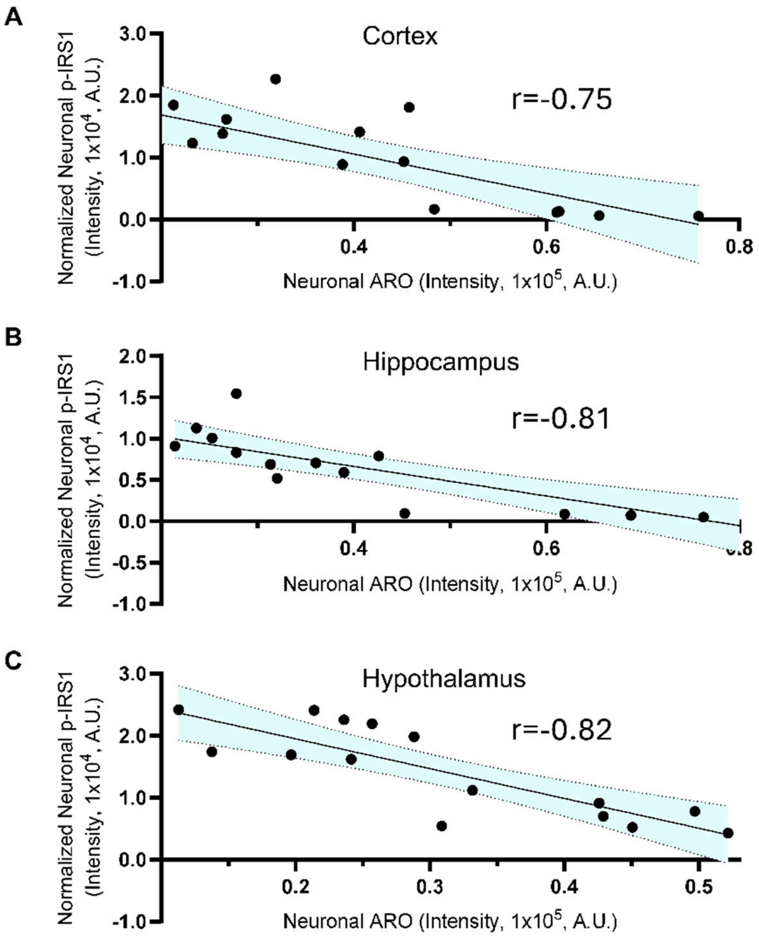

Figure S2. Correlation analysis of neuronal insulin resistance signaling and aromatase alterations in 5xFAD mice. (**A**) Linear relationship between normalized neuronal p-IRS1^{S616} signaling and neuronal aromatase intensities in the cortex of 5xFAD mice, with the correlation coefficient r value shown. (**B**) Correlation analysis of normalized neuronal p-IRS1^{S616} and aromatase intensities in hippocampal CA1 of 5xFAD mice, with correlation coefficient value r shown. (**C**) Correlation analysis of normalized neuronal p-IRS1^{S616} and aromatase intensities in 5xFAD hypothalamus, with r value shown and the 95% confidence interval indicated in the shaded area. $N = 15$. ARO, aromatase. A.U., arbitrary unit.

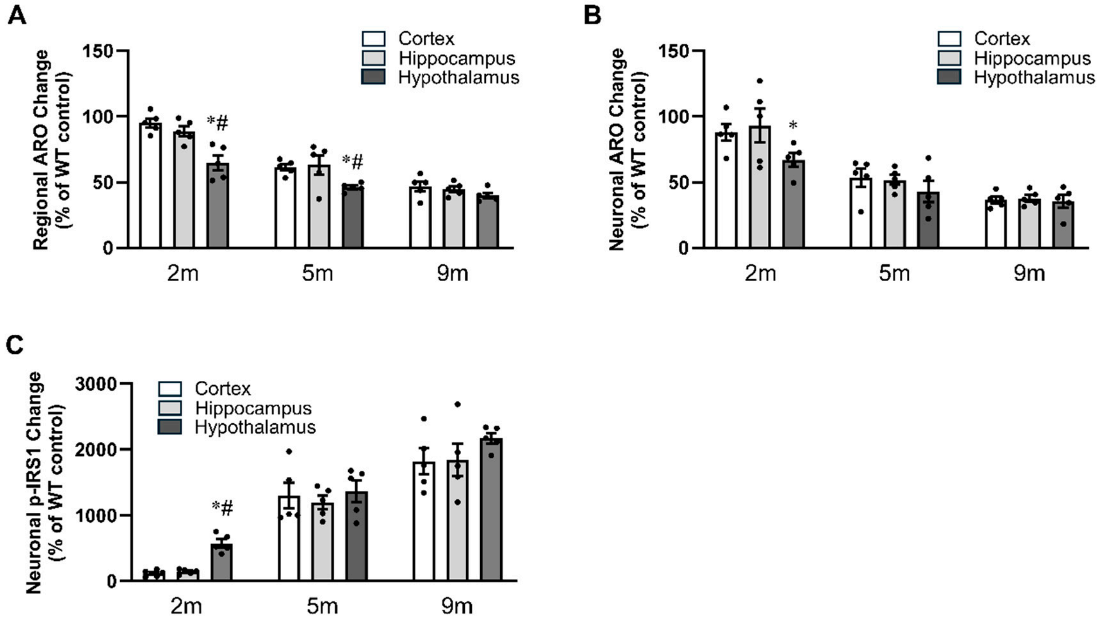

Figure S3. Comparisons of aromatase and p-IRS1 changes among different brain regions. (**A**) Analysis and comparisons of aromatase percentage of their own WT controls among cortex, hippocampus and hypothalamus of 2-month, 5-month and 9-month 5xFAD mice. (**B**) Percentage changes of neuronal aromatase among these brain regions at 2 months, 5 months and 9 months of ages. (**C**) Percentage changes of neuronal p-IRS1 among these regions at 2 months, 5 months and 9 months of ages. Values are means ± SEM from each group. $N = 5$. $^{*}P<0.05$ vs. Cortex, $^{\#}P<0.05$ vs. Hippocampus. ARO, aromatase. WT, wild-type.

Table S1. Summary of statistical values. Statistical values (F-values, Degrees of Freedom,

p-values) of the analysis using one-way ANOVA for the comparisons across WT control, 2-month, 5-month, 9-month 5xFAD mice were presented.

Figures		F-values	Degrees of Freedom (between-groups, within-groups)	p-values
Figure 1	Fluorescence Intensity of Whole Brain Aromatase	63.64	3, 16	<0.001
Figure 2	Intensity of ARO in Cortex	54.16	3, 16	<0.001
	Intensity of ARO in Hippocampus	29.29	3, 16	<0.001
	Intensity of ARO in Hypothalamus	48.44	3, 16	<0.001
Figure 3	Intensity of NeuN-positive ARO in Cortex	18.31	3, 16	0.002
	Intensity of NeuN-positive ARO in Hippocampus	31.59	3, 16	<0.001
	Intensity of NeuN-positive ARO in Hypothalamus	16.25	3, 16	<0.001
Figure 4	Intensity of GFAP-positive ARO in Cortex	25.57	3, 16	<0.001
	Intensity of GFAP-positive ARO in Hippocampus	34.11	3, 16	<0.001
	Intensity of GFAP-positive ARO in Hypothalamus	18.85	3, 16	0.001
Figure 5	Normalized Intensity of NeuN-positive p-IRS1(S616) in Cortex	37.63	3, 16	<0.001
	Normalized Intensity of NeuN-positive p-IRS1(S616) in Hippocampus	39.97	3, 16	<0.001
	Normalized Intensity of NeuN-positive p-IRS1(S616) in Hypothalamus	91.60	3, 16	<0.001
Figure S1	Intensity of NeuN-positive IRS1 in Cortex	7.781	3, 16	0.002
	Intensity of NeuN-positive IRS1 in Hippocampus	7.588	3, 16	0.002
	Intensity of NeuN-positive IRS1 in Hypothalamus	16.912	3, 16	<0.001
	Regional ARO Change – 2 months	13.032	2, 12	<0.001

Figure S3	Regional ARO Change – 5 months	4.319	2, 12	0.039
	Regional ARO Change – 9 months	1.638	2, 12	0.235
	Neuronal ARO Change – 2 months	2.505	2, 12	0.123
	Neuronal ARO Change – 5 months	0.762	2, 12	0.488
	Neuronal ARO Change – 9 months	0.098	2, 12	0.907
	Neuronal p-IRS1 Change - 2 months	43.375	2, 12	<0.001
	Neuronal p-IRS1 Change - 5 months	0.291	2, 12	0.753
	Neuronal p-IRS1 Change - 9 months	1.108	2, 12	0.362
